# K-factor image deshadowing for three-dimensional fluorescence microscopy

**DOI:** 10.1038/srep13724

**Published:** 2015-09-03

**Authors:** Tali Ilovitsh, Aryeh Weiss, Amihai Meiri, Carl G. Ebeling, Aliza Amiel, Hila Katz, Batya Mannasse-Green, Zeev Zalevsky

**Affiliations:** 1 Faculty of Engineering, Bar-Ilan University, Ramat-Gan 52900, Israel; 2 Department of Electrical and Computer Engineering, University of Utah, Salt Lake City, Utah, USA; 3 Department of Physics and Astronomy, University of Utah, Salt Lake City, UT; 4 Genetic Institute, Meir Medical Center, Kfar Saba, Israel; 5 Faculty of Life Sciences, Bar Ilan University, Ramat Gan, Israel

## Abstract

The ability to track single fluorescent particles within a three dimensional (3D) cellular environment can provide valuable insights into cellular processes. In this paper, we present a modified nonlinear image decomposition technique called K-factor that reshapes the 3D point spread function (PSF) of an XYZ image stack into a narrow Gaussian profile. The method increases localization accuracy by ~60% with compare to regular Gaussian fitting, and improves minimal resolvable distance between overlapping PSFs by ~50%. The algorithm was tested both on simulated data and experimentally.

Optical sectioning is a technique that enables 3D visualization of biological samples, by scanning the sample at different depths in the longitudinal direction. The Z-stack images are combined into a 3D image using computer post processing^1^. The resulting 3D images allow visualization of spatially resolved molecular scale details of the substructures in living cells. Because of the finite-lens aperture of the imaging system, microscopy techniques are diffraction limited^2^, making focal intensity distribution of any object that is below the Rayleigh criterion appear as a radial diffractive ring pattern with a point spread function (PSF)^3^,^4^.

Localization microscopy techniques such as (f)PALM^5^,^6^ and STORM^7^ exceed the diffraction limit and achieve super resolution. They are based on the sparse activation of individual fluorophores within a sample. The activated fluorophores are spatially well separated and can be imaged individually. By using the a priori knowledge of the PSF, the coordinates of each individual fluorophore can be determined with high accuracy, using localization algorithms^8^ such as non-linear least squares filtering^9^,^10^, and maximum likelihood estimation^11^. This activation and imaging process is repeated many times and the measured coordinates are plotted to generate a composite image^6^.

The localization precision depends on the accuracy of the PSF model that is being used. In 3D optical sectioning imaging, the PSF shape has an in-focus plane, and also out-of-focus contributions from other parts of the object. Due to the fact that the camera images are 2D, the localization in the lateral direction is relatively simple, based on the focal intensity distribution. However, localization in the axial direction is more complicated for two reasons. First, the intensity distribution has an axial symmetry that changes relatively slowly around the in-focus plain, making these shifts difficult to detect. Second, the axial position must be inferred from the defocused 2D intensity distributions, taking the complex dependence of the focal intensity distribution in the axial direction into account.

The PSF model has a major influence on the localization precision. The Gaussian function, which has a relatively low computational complexity, provides a good approximation for the in-focus plane, whereas for the out-of-focus planes, a more realistic profile is given by the Gibson and Lanni model^12^. The trade-off between choosing simplified PSF models and realistic models is time versus accuracy.

Other factors that degrade the accuracy are out-of-focus fluorescence, scattering, background noise and shot noise (especially where the photon count is low). Overlapping PSFs are a severe problem since they disrupt the localization process. Therefore, regions of interest (ROIs) exhibiting elongated (asymmetric) intensity profiles within a frame are often discarded from the sample set, effectively reducing the sampling density and increasing the image acquisition time^13^. This problem is more severe in the case of 3D imaging, due to the nature of the PSF in the axial direction, which has long tales that penetrate near-by emitters. In addition, in focused PSFs have a high probability to overlap with out of focus PSFs^14^.

3D localization methods include analysis of the diameter of the rings appearing in the defocused images^15^. Other methods are based on breaking the axial symmetry, as is done by the biplane^16^,^17^ and astigmatic detection^18^ methods. These techniques achieve lateral and axial resolutions of ~10 nm and ~30 nm respectively. However, they require more complicated optical systems, and complex post processing algorithms. In addition, the issue of overlapping PSFs must still be addressed. 2D techniques for overlapping emitter detection^19^,^20^ are much more complex in 3D.

In this paper we present a modified nonlinear image-factorization K-factor algorithm designed for the enhancement of different contrast levels within an image^21^. This algorithm sharpens the point source PSF, which provides two benefits. First, the 3D PSF shape is transformed into a 3D Gaussian profile, which simplifies the localization process and reaches the same high precision accuracy. Second, two overlapping PSFs become effectively separated, thus are able to be separately localized in 3D. The proposed algorithm is a computer post processing technique that can be applied to the raw data prior to emitter localization. In a previous paper, the algorithm was applied to 2D images^22^ showing improvement in localization precision or data acquisition time of ~40%. Here we present a modified version, which is applied to 3D localization.

## Theoretical Background

The 3D PSF of an aberration-free defocused imaging system with a finite lens aperture is given by an Airy function^23^ that expands with defocus. Each optical section includes both in-focus plane and out-of-focus contributions that are determined by the corresponding pupil function^24^. The ideal PSF function has circular symmetry about the z-axis, and mirror symmetry about the central xy-plane, and can be described by the Gibson and Lanni model that assumes an optical path that includes a biological sample, a cover slip layer and an immersion layer^25^. A mathematically simpler model is a Gaussian function^26^. The measured intensity is degraded by added photon shot-noise and background noise that is created mainly by out-of-focus and extraneous fluorescence and charge coupled device (CCD) readout noise. The Gaussian model for the intensity at (*x, y, z*) of a fluorescent particle located at (*x*_*0*_*, y*_*0*_*, z*_*0*_), is given by^27^:


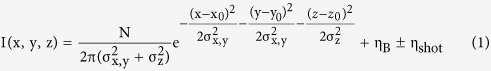


where *N* is the total number of collected photons during acquisition period, *σ*_*x,y*_ and *σ*_*z*_ are the standard deviation of the Gaussian, *η*_*B*_ is a Poisson distributed random variable with variance *N*_*b*_ that describes the background noise, and *η*_*shot*_ is a Poisson distributed random variable that describes the shot noise with a mean equal to the square root of the total intensity in each pixel^28^. In the absence of aberrations, the standard deviations will be given by^24^,^29^,^30^:


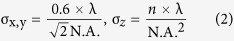


where N.A. is the numerical aperture of the objective, *λ* is the wavelength of the emitted light and *n* is the refractive index of the medium between the coverslip and the objective front lens element. The Gaussian model is a good approximation for confocal laser scanning microscopy (CLSM), however for wide-field and 3D PALM, a more realistic model must be used^26^.

## K-Factor Algorithm

The K-factor algorithm is an nonlinear image decomposition method^21^,^31^, that divides the image into a set of contrast based factors. The reconstructed image is the joint product of all harmonics in the decomposition. Here we demonstrate a modification of the original algorithm.

The K-factor transformation of an image *I(x, y)* is defined as:


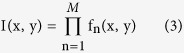


where *M* is the number of factors that reconstruct the image, and *f*_*n*_ are the different factors of the decomposition. The factors are given by:


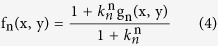


where *k*_*n*_ is the contrast depth parameter and its value varies between *0* and *1*. In the original decomposition we used, the parameter *k* was constant for all the different factors. However, varying *k* yields improved results, as will be discussed further on. *g*_*n*_ is a binary image computed as:


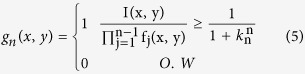


This is an iterative process. For each *n*, *g*_*n*_ is calculated as a binary image with pixel values that are either *1* or *0* according the threshold set by *k*_*n*_. Once *g*_*n*_ is calculated, *f*_*n*_ can be found. The next iteration will use the original image divided by the factor *f*_*n*_ as the threshold for the next iteration of *g*_*n*_. Due to the nature of the decomposition, when a fixed parameter k is used, the first few factors contain the details with the highest contrast in the image. These details are the desired image information along with some spatial noise. Higher order factors contain mostly spatial noise, together with some fine spatial information associated with low contrast levels. In a previous paper, we reported that the joint product of the original image and the first few factors of the decomposition, produces a sharper image, with data that is de-emphasized and reduced noise^22^. The choice of *k* controls the contrast depth of the factors. A high value close to one produces an image version with fine geometrical details of the image, but requires many factors. A value close to zero requires relatively few factors but produces large contrast steps and a spatially coarse version of the image.

Since the desired image information is usually concentrated mostly in the first few factors, the factors were assigned a high k value. The k-value was gradually reduced for the higher factors. In order to find an optimal set of parameters for the decomposition, an object consisting of two closely spaced Gaussian point sources was tested. The parameter *k* for each factor was allowed to vary, together with the number of factors M and the additive noise level of the image. The search was designed to maximize the height of the maxima of the PSFs, while minimizing the saddle between them, therefore maximizing the PSF peak to minimum saddle ratio. The optimal choice was found to be given by:


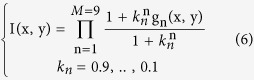


**Figure 1:**
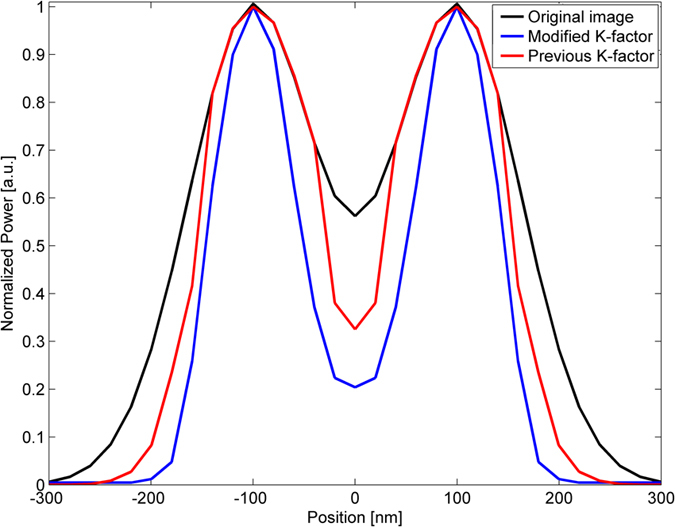
Image of two PSFs separated by a distance of *σ* = *200* *nm*. Original image (black), proposed K-factor transform (blue) and a comparison to the previous reported K-factor transform (red).

Figure 1 illustrates the influence of the proposed modified K-factor transform on a noiseless image containing two PSFs. The PSFs were created using Equation (1), with *σ*_*x*_ = *σ*_*y*_ = *200* *nm, σ*_*z*_ = *0* and the distance which the PSFs are separated by was *σ*_*x*_ = *σ*_*y*_ = *200* *nm*. In order to visualize the effect of the proposed technique on the obtained image, this illustration was performed in the absence of noise. The simulation conditions and the modelled optical system are described in detail, in the simulations section. It is compared to the previous algorithm with K-factor parameters *k* = *0.9*, *M* = *48*, *h* = *8*. In the proposed technique, the saddle between the two PSFs was reduced by a factor of *2.2* compared to a reduction of *1.5* for the previous algorithm. In addition, the *σ* of the PSF was reduced by *50%*. This reduction enables the detection of overlapping emitters with reduced separation. Furthermore, here, only *9* factors are needed for the decomposition, compared to *48* in the previous work.

The proposed technique can be added to the conventional PALM workflow. The 3D image acquisition technique begins with the capturing of a set of z-stack images of the sample. The images are then processed with the K-factor algorithm. Next, a localization routine using methods of single emitter fitting produces the 3D emitter positions. The final super-resolved image is the linear combination of all emitters coordinates.

## Simulations

**Figure 2:**
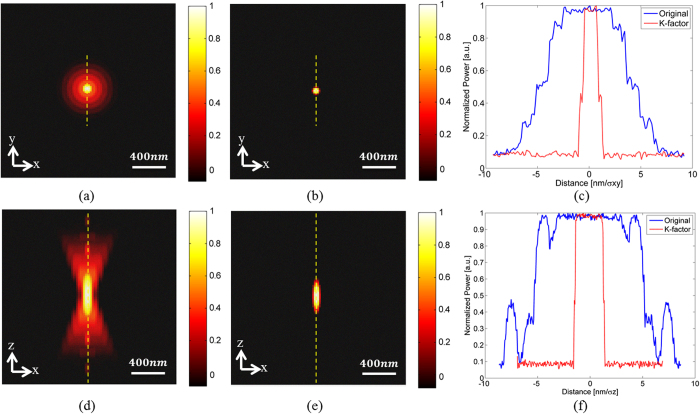
Simulated profiles of the PSF shot noise and N_b_ = 5. (**a**) lateral profile of the PSF; (**b**) K-factor algorithm applied to (**a**); (**c**) Line profiles of the dashed lines in (**a**,**b**); (**d**) axial profile of the PSF; (**e**) K-factor algorithm applied to (**d**); (**f**) Line profiles of the dashed lines in (**d**,**e**).

Monte-Carlo (MC) simulations were used in order to generate simulated data sets with emitters at different lateral and axial positions in each set. In these simulations, the model was a fluorescence point source emitting at *λ* = *540* *nm* that was imaged through an objective lens onto a CCD camera. The model parameters were chosen to correspond to an optical system consisting of a *63x/NA* = *1.4* oil immersion objective lens, imaged though a 1.0x relay lens onto a CCD sensor array with *6.45μm* *×* *6.45* *μm pixels*, which translates to *102* *nm* *×* *102* *nm* in the object plane with the *63x* objective. The z-step size was *Δz*_*i*_ = *200* *nm*. Background noise was introduced by adding a Poisson distributed random value with the parameter *N*_*b*_, that can be measured experimentally and therefore is considered to be known^32^. In our simulations we choose an average sized background noise of *N*_*b*_ = *5* photons. Shot noise was added as a Poisson process with an expected value which corresponds to the noiseless pixel values and a standard deviation (STD) that equals the square root of the value of each pixel. The value for *σ*_*x,y*_ and *σ*_*z*_ for the given imaging parameters was calculated from Equation (2) to be *167* *nm* and *413* *nm* respectively. Figure 2 is the simulated lateral and axial PSF profiles with added shot noise (N = 5000) and N_b_ = 5. Figure 2(a) is a simulation of the lateral profile of the PSF and Figure 2(d) is a simulation of the axial profile of the PSF. When the K-factor algorithm is applied to the PSF, the areas with highest contrast in the PSF are enhanced and low level regions are discarded. The resulting PSF shape is well approximated by a Gaussian, both in the lateral (Fig. 2b) and the axial (Fig. 2e) directions. In order to compare the original and K-factor processed images, the line profiles of the yellow dashed lines in Fig. 2(a,b) is presented in Fig. 2(c), and the line profiles of the yellow dashed lines in Fig. 2(d,e) is presented in Fig. 2(f).

The transform of the PSF into a Gaussian shape improves the localization precision of each emitter. In order to test this assumption, the raw simulated data set of z-stack images of a single emitter with added shot noise and background noise (*N*_*b*_ = *5* photons), and the K-factor filtered data, were processed with a least square localization routine that was implemented in MATLAB. The position of the fluorophore and the number of arriving photons *N* were allowed to vary. The initial z position of the emitter was chosen to be the most focused image in the set. The 3-D neighborhood around each local maximum was fit with both the Gibson and Lanni model, and a Gaussian model of the form of:


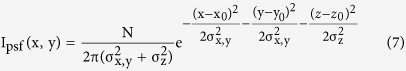


Where *N, σ*_*x,y*_, *σ*_*z*_, *x*_*0*_, *y*_*0*_ and *z*_*0*_ are all fit parameters. The algorithm’s initial coordinates were the local maximum. The fit was carried out by taking *9* *×* *9* *×* *9* pixels around the initial location^25^. The fit produces the best estimate of the position of the fluorophore and was repeated for 1000 MC iterations yielding a set of *L* = *1000* localization positions (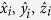) that can be compared with the known original position (*x*_*i*_*, y*_*i*_*, z*_*i*_). The root mean square (RMS) localization error was computed for both the raw and K-factor filtered simulated data:


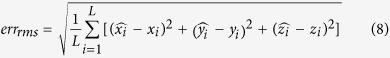


**Figure 3:**
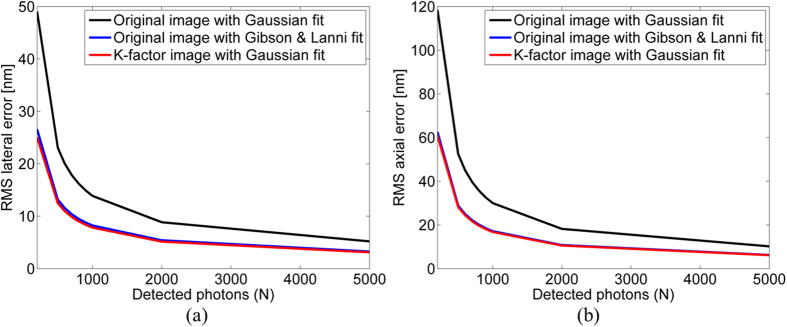
Error in localization as a function of detected photons with added shot noise and background noise (*N*_*b*_ = *5*). The simulation contained and *1000* Monte-Carlo iterations. (**a**,**b**) are the lateral and axial RMS localization error, respectively. Gaussian fitting applied to raw data is presented in the black line. Gibson & Lanni fitting applied to the raw data is presented in the blue line and Gaussian fitting applied to the raw data that underwent processing with the K-factor algorithm is the red line.

Figure 3 compares the RMS localization error of the fitting process for the lateral direction (Fig. 3a) and for the axial direction (Fig. 3b) as a function of detected photons with added shot noise and background noise (*N*_*b*_ = *5*). Gaussian fitting applied to raw data is presented in the black line. Gibson & Lanni fitting applied to the raw data is presented in the blue line and Gaussian fitting applied to the raw data that underwent processing with the K-factor algorithm is the red line. As was expected, for the raw data, the Gibson and Lanni model yielded good results for both the lateral and axial directions, whereas for the processed data, the Gaussian model yielded similar results. These results are more accurate by a factor of two with compare to a Gaussian fitting of the raw data. This is due to the K-factor algorithm’s ability to transform the 3D PSF shape to fit that of a Gaussian.

With regard to the photon number, as can be seen in Fig. 3, an increase in *N* lowers the RMS error. This is due to the shot noise that is a Poisson random process with a rate that depends on the total number of photons detected, and is proportional to *√N* and therefore is reduced with higher *N*^33^.

Another advantage of the proposed technique is that it effectively narrows the PSF width. When the width is smaller, overlapping PSF can be identified and localized separately. Since frames that contain overlapping PSFs are usually discarded, the ability to detect them will decrease image acquisition time. This problem is more pronounced in 3D imaging, since the contribution from each emitter is spread over a larger volume. In order to determine the minimal distance at which two emitters can be resolved, the distance between two emitters for both the lateral and axial axes and the added shot noise and background noise parameters were varied. The results are summarized in Table 1.

**Figure 4:**
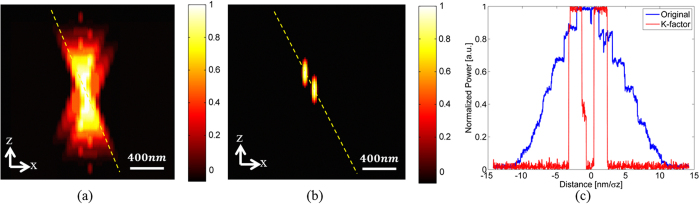
(**a**) axial profile of two PSFs separated by 450 nm; (**b**) K-factor algorithm applied to (**a**); (**c**) Line profiles of the dashed lines in (**a**,**b**).

It is seen that for the lateral axis, the K-factor reduces the minimum distance required to resolve two point emitters by more than a factor of two, and reaches *250* *nm*.The improvement is even greater in the axial axis, due to the widening of the PSF in the z-direction. The axial PSFs profile for the distances that are presented in Table 1 is shown in Fig. 4 with N = 5000, N_b_ = 5. Figure 4(a) is the original axial profile. Figure 4(b) is the K-factor algorithm applied to Fig. 4(a,c) is line profiles of the dashed lines in Fig. 4(a,b). In the original image, the two PSFs were undistinguishable, whereas with the K-factor processed image, there was a distinct separation between the two PSFs, as well as the reshaping of the PSF into a Gaussian shape.

## Materials and Methods

Peripheral-blood lymphocytes (previously extracted from whole blood) were placed on a glass slide, and the telomere labeling was carried out using a Dako K5326 Telomere PNA FISH Kit/Cy3 (Dako, Carpinteria, Canada). Briefly, the cells were fixed and stripped on the slide as per the protocol, and hybridization was done with the HYBrite system (Abbot Laboratories, Des Plaines, IL, USA). The slides were then counterstained with DAPI and mounted using DAPI II counterstain medium (Abbot Laboratories, Des Plaines, IL, USA). The slides were then covered with a #1.5 glass coverslip and sealed with nail-polish. All experiments which involved human blood samples were carried out in accordance with the approved guidelines and regulations, and the experimental protocols were approved by the Meir Hospital Institutional Committee for Ethics. Informed consent was obtained from all study participants.

Z-stacks of the FISH labeled telomeres were acquired using a fully automated Nikon TE2000E inverted fluorescence widefield microscope, through a 60x/NA = 1.4 oil immersion objective. The excitation source was an xCite 120 W metal halide source (Excelitas Technologies Corp, Waltham, MA, USA), and a narrowband Cy3 filter set (#41003, Chroma Technology, Bellows Falls, Vermont USA) was used. Images were acquired with a Retiga 2000 R cooled CCD camera (QImaging, Surrey, BC, Canada) with *7.4* *μm* × *7.4* *μm* pixel size. The system was controlled with Nikon’s NIS elements software. Multiple fields were acquired automatically, and in each field, a Z-stack of *17* slices at *200* *nm* spacing was acquired. The multifield ND2 images were preprocessed using the Fiji distribution of ImageJ^34^. The raw ND2 files were read using the Bioformats plugin^35^. The desired field and channel was extracted, and the image stack was cropped to an area of *135* *×* *165* pixels, selected to include one cell nucleus, in order to reduce computation time, with an effective pixel size of *117.5* *nm*. The cropped image stack was saved as a TIFF image sequence for further downstream processing in MATLAB.

The K-factor algorithm was implemented in MATLAB (version 2012b, MathWorks, Natick, MA, USA). The program was run on a HP Compaq Elite 8300 Microtower PC with Windows 7 Professional 64 bit operation system, Intel® Core™ i5-3470 processor, 3.20 GHz, 12 GB RAM.

## Experimental Results

**Figure 5:**
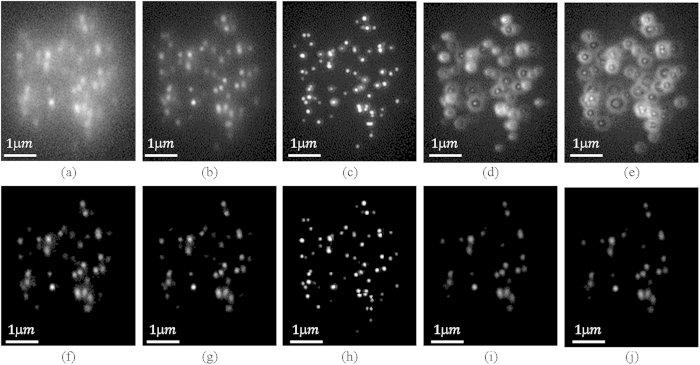
Experimental results of a z-stack images. The upper row (**a**–**e**) are a sequence of images with Δz = 400 nm between fames. The in-focus image is (**c**). The lower row (**f**–**j**) are the original frames after the application of the K-factor algorithm.

**Figure 6:**
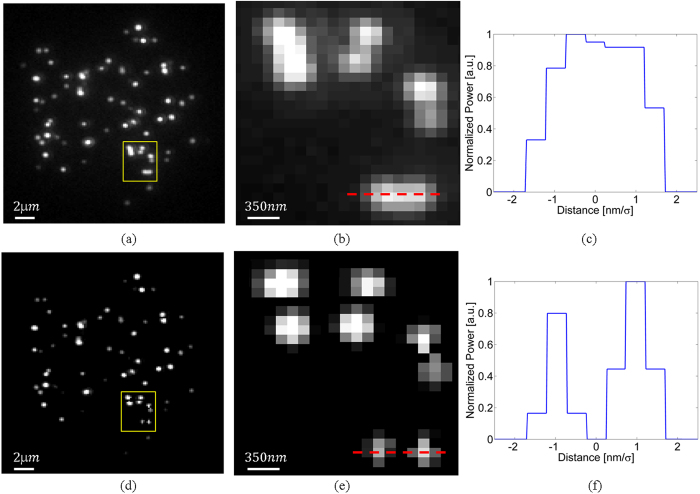
Experimental results in the lateral direction. (**a**) The unprocessed focused frame from the z-stack measurements. Marked regions are areas with closely spaced emitters. (**b**) The magnification of the marked areas in (**a**). (**d**) the K-factor processed image presented in (**a**,**e**) is the magnification of the marked area. (**c,f**) show the cross section of the dashed line that passes through the center of the overlapping two emitters in the lower part of the image in (**b**,**e**) respectively.

The raw z-stack FISH labeled telomeres images (described in the methods section) were filtered with the K-factor algorithm, after which a LS localization procedure was applied in MATLAB. The raw data was fitted to a Gibson and Lanni PSF profile, while the K-factor processed images were fitted to a Gaussian profile. The fluorescence data typically included dozens emitting of telomeres simultaneously. Figure 5(a–e) presents a sequence of XY images at two planes apart, with *Δz* = *400* *nm* between frames. Figure 5(c) is the most focused image, as determined by eye. Figure 5(f–j) are the frames after the application of the K-factor algorithm. The obtained profile of the raw data PSF, matched the one expected from Fig. 2(d), and is widening as the defocus grows. For the K-factor processed images, the PSF profile matches Fig. 2(e) and is narrowing as the defocus grows. The localization routine was done both on the raw data images and on the K-factor processed images. The focused images were chosen as the initial z coordinates for the localization routine, and the 3D neighborhood around each local maxima were fitted using the LS algorithm with the corresponding PSF shape. The unprocessed focused frame from the z-stack measurements is shown in Figure 6(a). Overlapping emitters can be seen as spots with a larger radius. One such region of closely spaced molecules is marked by a yellow frame, which is magnified in Figure 6(b). By applying the K-factor algorithm on each individual frame (Fig. 6d), areas with overlapping molecules became distinguishable, as can be seen in the magnified area (Fig. 6e). Figure 6(c,f) show the cross section of the dashed line that passes through the center of the overlapping two emitters.

**Figure 7:**
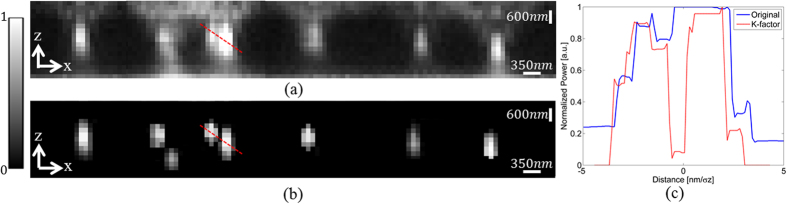
Experimental results in the axial direction. (**a**) an unprocessed frame from the z-stack measurements. (**b**) the K-factor processed image presented in (**a**). (**c**) Line profiles of the red dashed lines in (**a**,**b**).

The PSFs recorded in Fig. 6(b) are longitudinally extended and lacks any form of symmetry, therefore in the absence of serious optical aberrations or distortions, it clearly originates from multiple emitters, and therefore the localization process is not applicable in this case. In the K-factor processed image (Fig. 6e), these PSFs were localized as two distinct emitters. This is due to the reduction in the PSF width, which reduced the saddle between the PSFs and enabled their localization. The same effect occurs in the axial direction, as can be seen in the axial direction as can be seen in Fig. 7. Each pixel in the axial direction corresponds to *200* *nm*, whereas in the lateral direction it is *117* *nm*.

The Gibson and Lanni PSF profile is clearly seen in Fig. 7(a), whereas it becomes a Gaussian shape in the K-factor processed image Fig. 7(b,c) is the line profiles of the red dashed lines in Fig. 7(a,b). In the original image there are overlapping PSFs that appear as large spots that resolves into two separate PSFs in Fig. 7(b). Furthermore, in the processed image, background noise is reduced which makes the background appear totally black. An important remark is the existence of Spherical aberration when performing 3D imaging of thick samples. This aberration is due to the refractive index mismatch between the imaging medium and the objective, which effectively increasing the apparent z position of each emitter. Such aberration is clearly present in the data in the defocussed images of Fig. 5(a) compared to Fig. 5(e). There are numerous ways to overcome this aberration^36^. One is to use an imaging medium with a higher refractive index. The other is to apply the localization procedure only on emitters that are below the focal plane. Alternatively, there are objective lenses available with correction ring for spherical aberration^37^. Here, when applying the K-factor algorithm to the PSFs image, the result is a Gaussian shape. Simulations show that the effect of the spherical aberrations with the proposed technique may cause a shift of the Gaussian shape which causes a constant drift error in the z position throughout the entire image. Since this is a constant drift, it does not affect the obtained 3D reconstruction and can be corrected using a rescaling factor, without any decrease in the localization precision.

**Figure 8:**
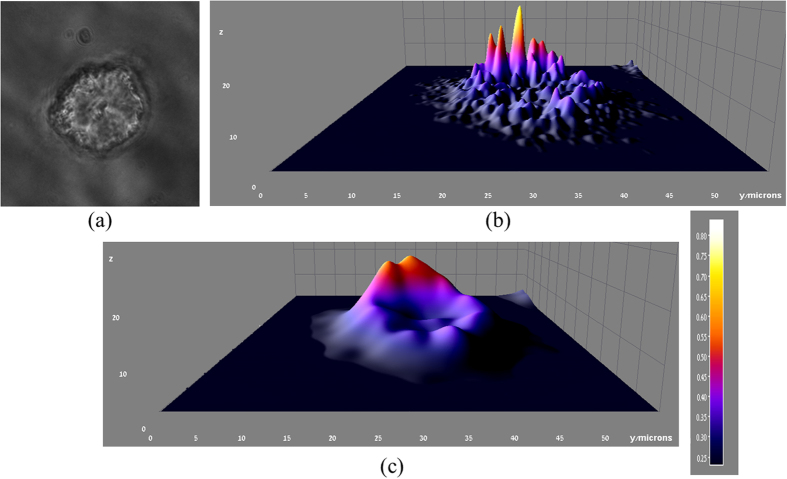
3D reconstruction of a single A431 cell out of a 2D z-stack set of images. (**a**) a phase image of the cell. (**b**) The cell’s 3D image using the original z-stack. (**c**) The cell’s 3D image using the K-factor processed z-stack set. The scale bar is identical for both (**b**,**c**).

In order to calculate the localization precision at all directions, the Gaussian localization routine was applied to *100* different emitters in each of two sets, and the standard deviation of particles coordinates was calculated for *20* repeated experiments. The standard deviation σ_x,y_ of the raw data was *5.2* *nm*, which corresponds to a resolution of (FWHM = *2.35σ*) *12.22* *nm*. The K-factor algorithm applied on the row data yielded a standard deviation of *2.3* *nm*, which corresponds to resolution of *5.4* *nm*, an improvement of ~*60%*. With the same analysis, the axial resolution was *86.15* *nm* with compare to *27.2* *nm* in the processed data which is an improvement of ~*70%*. The Gibson and Lanni localization routine applied to the raw data yielded resolution of *28.6* *nm*. This relatively low resolution stems from the sampling in the z-direction that was done with steps of *200* *nm*. A smaller Z-step would increase the resolution. In addition, for each set, the number of detected PSFs was measured and averaged. The results show an increase of 50% in the density of the PSFs with the K-factor algorithm, thus enables an increase in the activated fluorophore density inside the sample. As a last step to prove the viability of the proposed technique to a 3D image of a biological sample, samples of human epidermoid carcinoma cell line, A431^38^, that were loaded with *20* *nm* spheres GNPs immobilized on a coverslip, using a known protocol were used^39^,^40^. Each sample was illuminated using a green laser at *532* *nm* (Photop DPGL-2100 F). The Z-stack set of images of the scattered light from the sample was acquired using a fully automated Nikon TE2000E inverted fluorescence widefield microscope, through a 40x/NA = 0.6 long working distance air objective. Images were acquired with a Retiga 2000 R cooled CCD camera (QImaging, Surrey, BC, Canada). Multiple fields were acquired automatically, and in each field, a Z-stack of *40* slices at *100* *nm* spacing was acquired. The multifield ND2 images were preprocessed using the Fiji distribution of ImageJ^34^. The raw ND2 files were read using the Bioformats plugin^35^, where they were cropped to contain a single cell. The obtained 2D z-stack set is merged in order to create a 3D image of the cell using post processing and is presented in Fig. 8. Figure 8(a) is a phase image of the single cell. Figure 8(b) is the obtained 3D image of the cell using the original z-stack. The K-factor algorithm was applied to each z-stack frame individually and the entire K-factor processed set was used to create the 3D cell’s image (Fig. 8c). The scale bar is identical for both Fig. 8(b,c). The original 3D image contains sharp peaks due to the PSF shape in the axial direction, which broadens with defocus, whereas in the K-factor 3D processed image the result is smoother without sharp edges, due to the reshaping of the PSF into a Gaussian shape. Thus, the technique is applicable to 3D data.

## Summary and Conclusions

This paper presents the use of the K-factor algorithm for enhanced performance of 3D localization microscopy techniques. There are three major advantages of the K-factor algorithm over the used Gibson-Lanni model. For the case of a single PSF, the Gibson-Lanni model is very noise sensitive, which results in an increased localization error, whereas the K-factor algorithm is a noise reduction technique, thus is much less sensitive to noise. In addition, the computational complexity involving the localization using the Gibson-Lanni model is very high and time consuming. The fit of the PSF to a Gaussian profile is fast and simple, however results in an enlarged localization error. The K-factor algorithm reshapes the PSF into a Gaussian profile, enabling its localization using a regular Gaussian shape with the same precision as the Gibson-Lanni model and at a shorter period. For the case of closely spaced PSFs, the Gibson-Lanni model fails to perform their localization, whereas the K-factor algorithm separates the PSFs, which enables their localization. The proposed approach is generic and can be applied to any 3D microscope configuration, where the ability to use Gaussian fitting with high accuracy on 3D data can facilitate the computational complexity, hence reduce the processing time required for the generation of the 3D superresolved image.

## Additional Information

**How to cite this article**: Ilovitsh, T. *et al*. K-factor image deshadowing for three-dimensional fluorescence microscopy. *Sci. Rep*. **5**, 13724; doi: 10.1038/srep13724 (2015).

## Figures and Tables

**Table 1:** Minimal distance at which two emitters can be resolved by both the standard LS method and the proposed K-factor.

	Standard LS Method	K-factor
Lateral Axis [nm]	550	250
Axial Axis [nm]	1100	450

## References

[b1] Conchello J. A. & Lichtman J. W. Optical sectioning microscopy. Nat. Methods 2, 920–31 (2005). 16299477 10.1038/nmeth815

[b2] Dempster W. T. Principles of microscope illumination and the problem of glare. J. Opt. Soc. Am. 34, 695–710 (1944).

[b3] Rayleigh L. X. V. On the theory of optical images, with special reference to the microscope. London, Edinburgh, Dublin Philos. Mag. J. Sci. 42, 167–195 (1896).

[b4] Abbe E. Beitrage zur theorie des mikroskops und der mikroskopischen Wahrnehmung. Arch. für mikroskopische Anat. 9, 413–418 (1873).

[b5] Betzig E. . Imaging intracellular fluorescent proteins at nanometer resolution. Science. 313, 1642–1645 (2006). 16902090 10.1126/science.1127344

[b6] Hess S. T., Girirajan T. P. K. & Mason M. D. Ultra high resolution imaging by fluorescence photoactivation localization microscopy. Biophys. J. 91, 4258–72 (2006). 16980368 10.1529/biophysj.106.091116 PMC1635685

[b7] Rust M. J., Bates M. & Zhuang X. Sub-diffraction-limit imaging by stochastic optical reconstruction microscopy (STORM). Nat. Methods 3, 793–795 (2006). 16896339 10.1038/nmeth929 PMC2700296

[b8] Abraham A., Ram S., Chao J., Ward E. & Ober R. Quantitative study of single molecule location estimation techniques. Opt. Express 17, 23352–73 (2009). 20052043 10.1364/OE.17.023352 PMC2813811

[b9] Bobroff N. Position measurement with a resolution and noise-limited instrument. Rev. Sci. Instrum. 57, 1152 (1986).

[b10] Ober R. J., Ram S. & Ward E. S. Localization accuracy in single molecule microscopy. Biophys. J. 86, 1185–200 (2004). 14747353 10.1016/S0006-3495(04)74193-4 PMC1303911

[b11] Shepp L. & Vardi Y. Maximum likelihood reconstruction for emission tomography. IEEE Trans. Med. Imaging 1, 113–22 (1982). 18238264 10.1109/TMI.1982.4307558

[b12] Gibson S. F. & Lanni F. Experimental test of an analytical model of aberration in an oil-immersion objective lens used in three-dimensional light microscopy. J. Opt. Soc. Am. A 9, 154 (1992). 1738047 10.1364/josaa.9.000154

[b13] Henriques R., Griffiths C., Hesper Rego E. & Mhlanga M. M. PALM and STORM: unlocking live-cell super-resolution. Biopolymers 95, 322–31 (2011). 21254001 10.1002/bip.21586

[b14] Chao J. & Ram S. A 3D resolution measure for optical microscopy. Int. Symp. Biomed. Imaging From Nano to Macro 1115–1118 (2009). 10.1109/ISBI.2009.5193252 PMC2814427 20126419

[b15] Prabhat P., Ram S., Sally Ward E. & Ober R. J. Simultaneous imaging of different focal planes in fluorescence microscopy for the study of cellular dynamics in three dimensions. IEEE Trans. Nanobioscience 3, 237–242 (2004). 15631134 10.1109/tnb.2004.837899 PMC2761735

[b16] Juette M. F. . Three-dimensional sub-100 nm resolution fluorescence microscopy of thick samples. Nat. Methods 5, 527–9 (2008). 18469823 10.1038/nmeth.1211

[b17] Shtengel G. Interferometric fluorescent super-resolution microscopy resolves 3D cellular ultrastructure. PNAS 106, 3125–3130 (2009). 19202073 10.1073/pnas.0813131106 PMC2637278

[b18] Huang B., Wang W., Bates M. & Zhuang X. Three-dimensional super-resolution imaging by stochastic optical reconstruction microscopy. Science. 319, 801–813 (2008). 10.1126/science.1153529 PMC2633023 18174397

[b19] Mukamel E., Babcock H. & Zhuang X. Statistical deconvolution for superresolution fluorescence microscopy. Biophys. J. 102, 2391–400 (2012). 22677393 10.1016/j.bpj.2012.03.070 PMC3353000

[b20] Huang F., Schwartz S. L., Byars J. M. & Lidke K. Simultaneous multiple-emitter fitting for single molecule super-resolution imaging. Biomed. Opt. Express 2, 1377–93 (2011). 21559149 10.1364/BOE.2.001377 PMC3087594

[b21] Johnson J. L. & Taylor J. R. Image factorization: a new hierarchical decomposition technique. Opt. Eng. 38, 1517–1523 (1999).

[b22] Ilovitsh T. . Improved localization accuracy in stochastic super-resolution fluorescence microscopy by K-factor image deshadowing. Biomed. Opt. Express 5, 244–58 (2013). 24466491 10.1364/BOE.5.000244 PMC3891336

[b23] Juskaitis R. in Handb. Biol. Confocal Microsc. (ed. Pawley J. B. ) 239–250 (Springer, 2006).

[b24] Stokseth P. Properties of a defocused optical system. JOSA 1002, 1314–1321 (1969).

[b25] Kirshner H., Aguet F., Sage D. & Unser M. 3-D PSF fitting for fluorescence microscopy: implementation and localization application. J. Microsc. 249, 13–25 (2013). 23126323 10.1111/j.1365-2818.2012.03675.x

[b26] Zhang B., Zerubia J. & Olivo Marin J. C. Gaussian approximations of fluorescence microscope point-spread function models. Appl. Opt. 46, 1819–1829 (2007). 17356626 10.1364/ao.46.001819

[b27] Thompson R. E., Larson D. R. & Webb W. W. Precise nanometer localization analysis for individual fluorescent probes. Biophys. J. 82, 2775–83 (2002). 11964263 10.1016/S0006-3495(02)75618-X PMC1302065

[b28] Janesick J. R. Scientific Charge Coupled Devices. SPIE Press monograph, 605–419 (2001).

[b29] Waters J. C. Accuracy and precision in quantitative fluorescence microscopy. J. Cell Biol. 185, 1135–48 (2009). 19564400 10.1083/jcb.200903097 PMC2712964

[b30] Born M. & Wolf E. Principles of optics. Pergamon Oxford (Ed.) (Cambridge University Press, 1999).

[b31] Johnson J. L. & Taylor J. R. K-Factor Image Factorization. in AeroSense’99. Int. Soc. Opt. Photonics 3715, 166–174 (1999).

[b32] Snyder D. L., Helstrom C. W., Lanterman A. D., Faisal M. & White R. L. Compensation for readout noise in CCD images. JOSA A 12, 272–283 (1995).

[b33] Ghosh R. N. & Webb W. W. Automated detection and tracking of individual and clustered cell surface low density lipoprotein receptor molecules. Biophys. J. 66, 1301–18 (1994). 8061186 10.1016/S0006-3495(94)80939-7 PMC1275851

[b34] Schindelin J. . Fiji: an open-source platform for biological-image analysis. Nat. Methods 9, 676–82 (2012). 22743772 10.1038/nmeth.2019 PMC3855844

[b35] Linkert M. . Metadata matters: Access to image data in the real world. J. Cell Biol. 189, 777–782 (2010). 20513764 10.1083/jcb.201004104 PMC2878938

[b36] Huang B., Jones S. A., Brandenburg B. & Zhuang X. Whole cell 3D STORM reveals interactions between cellular structures with nanometer-scale resolution. Nat. Methods 5, 1047–1052 (2009). 19029906 10.1038/nmeth.1274 PMC2596623

[b37] Cella Zanacchi F. . Live-cell 3D super-resolution imaging in thick biological samples. Nat. Methods 8, 1047–1049 (2011). 21983925 10.1038/nmeth.1744

[b38] Masui H., Kawamoto T., Sato J. & Wolf B. Growth inhibition of human tumor cells in athymic mice by anti-epidermal growth factor receptor monoclonal antibodies. Cancer Res. 44, 1002–1007 (1984). 6318979

[b39] Reuveni T., Motiei M., Romman Z., Popovtzer A. & Popovtzer R. Targeted gold nanoparticles enable molecular CT imaging of cancer: an *in vivo* study. Int. J. Nanomedicine 6, 2859–2864 (2011). 22131831 10.2147/IJN.S25446 PMC3224712

[b40] Ilovitsh T., Danan Y., Meir R., Meiri A. & Zalevsky Z. Cellular imaging using temporally flickering nanoparticles. Sci. Rep. 5, 1–6 (2015). 10.1038/srep08244 PMC4316156 25650019

